# Alloferon-1 ameliorates acute inflammatory responses in λ-carrageenan-induced paw edema in mice

**DOI:** 10.1038/s41598-022-20648-z

**Published:** 2022-10-06

**Authors:** Xiangrui Zhang, Vladimir Retyunskiy, Shuai Qiao, Ye Zhao, Chi-Meng Tzeng

**Affiliations:** 1 School of Pharmaceutical Sciences, Nanjing Tech University, Nanjing, 211800 China; 2 School of Pharmaceutical Sciences, Xiamen University, Xiamen, 361005 China; 3 Translational Medicine Research Center-Key Laboratory for Cancer T-Cell Theragnostic and Clinical Translation, School of Pharmaceutical Sciences, Xiamen University, Xiamen, 361005 China; 4 Xiamen Chang Gung Hospital Medical Research Center, Xiamen, 361005 China

**Keywords:** Pharmacology, Target identification

## Abstract

Alloferon-1 have been proposed as an effective peptide to enhance antitumoral immunity, antiviral defense and anti-inflammatory activity. This work aimed to assess anti-inflammatory effects of alloferon-1 against acute inflammation and histopathological deformations in λ-carrageenan-induced paw edema in mice. Systemic pretreatment with alloferon-1 (22.0 mg/kg) intraperitoneally injected mice showed a significant reduction in paw thickness and vascular permeability. Alloferon-1 prevented λ-carrageenan-evoked exudation and the neutrophil influx to the mouse pleura and the neutrophil migration into carrageenan-stimulated mouse air pouches based on the histopathological changes in the paw tissues. Administration of alloferon-1 also suppressed the expression of the inflammatory cytokines in the inflamed paw tissues such as tumor necrosis factor-α (TNF-α), monocyte chemoattractant protein 1 (MCP1), interleukin-5 (IL-5), etc. detected by Luminex liquid chip. Collectively, the present study provides evidences for the marked anti-inflammatory effects of alloferon-1 which might represent new therapeutic options for the treatment of acute inflammatory diseases.

## Introduction

Inflammation refers to the defense response against allergens, toxic substances, infections or cell damages, leading to the occurrence of redness, swelling, asthma, fever, pain and other symptoms in the affected site^1–3^. Inflammation can be divided into acute inflammation and chronic inflammation. Acute inflammation is temporary and can be restored into normal tissue homeostasis by dispensing with the inciting stimulus. However, persistence acute inflammatory responses usually leads to rheumatoid arthritis, atherosclerosis, Alzheimer’s disease and bowel disease^4^. Currently, prescribed anti-inflammatory drugs including steroidal anti-inflammatory drugs, non-steroidal anti-inflammatory drugs (NSAIDs) and antibacterial drugs are commonly applied for the treatment of inflammation. Although often accompanied by mild or moderate adverse reactions, long-term use of large doses of these drugs have been correlated with serious health problems such as gastrointestinal bleeding and nephrotoxicity^8^. Therefore, it is important to understand the mechanisms of acute and chronic inflammation, and in particular to explore inhibitory intervention drugs to decline the immunological reactivity.

Antimicrobial peptides (AMPs), widely distributed in the biosphere, refer to a kind of conservative peptide molecules with antimicrobial activity. As a group of naturally occurring AMPs, alloferons have been proposed as effective peptide compounds to enhance antitumor immunity and antiviral defense. Alloferons are members of cytokine-like peptide family that were originally derived from a hemolymph of bacteria challenged maggots of the blow fly Calliphora vicina Diptera^5^. Two peptides of 13 and 12 amino acids, which stimulate in vitro natural cytotoxicity of mouse spleen lymphocytes and human blood mononuclear cells, were isolated and their amino acid sequences were identified as HIS-GLY-VAL-SER-GLY-HIS-GLY-GLN-HIS-GLY-VAL-HIS-GLY (alloferon-1) and GLY-VAL-SER-GLY-HIS-GLY-GLN-HIS-GLY-VAL-HIS-GLY (alloferon-2)^5^. Alloferon-1 reveals a capacity to stimulate the activity of natural killer (NK) cells and the synthesis of IFN in animal and human models, thereby exhibiting anti-tumoral and anti-viral capabilities^6^. Recently, it was also reported that alloferon-1 effectively reduces the production of pro-inflammatory cytokines, such as interleukin 5 (IL-5), interleukin 6 (IL-6), and interleukin 17 (IL-17) in ovalbumin-induced asthma^7^. Analogously in UVB-induced skin inflammation, alloferon-1 potently decreases the generation of pro-inflammatory cytokines, such as IL-6, interleukin 8 (IL-8), and tumor necrosis factor-α (TNF-α)^8^. In addition, alloferon-1 attenuates dextran sulfate sodium-induced enteritis by downregulation of IL-6 and TNF-α^9^. Based on these immune-modulating effects of alloferon-1, in the present study, we investigated the anti-inflammatory effect of alloferon-1 in λ-carrageenan induced paw edema in mice.

## Materials and methods

### Animals and reagents

The study was approved by Ethics Committee of Nanjing Tech University. Mice husbandry and disposal were according to the compliance with Institutional Animal Care and Use Committee (IACUC) guidelines of Nanjing Tech University for laboratory animal use and all methods were in compliance with the ARRIVE guidelines. Specific pathogen-free (SPF) male BALB/c mice (body weight 20–25 g) of 8–9 weeks of age were procured from Nanjing Junke Bioengineering Corporation, China. All mice were kept under controlled environmental conditions (25 ± 3 °C, 50–60% humidity and a 12 h light–dark cycle) and maintained with ad libitum access to standard diet and water. Except for drinking water, all mice were food deprived for 12 h before the start of experiments. All the experimental animals were fed, and all the operations were performed by the same researcher. No animals died during the experiments. At the end of experiments, mice were expertly euthanized by cervical dislocation method for sample collection.

Alloferon-1 (purity > 98.62%) was supplied by Leon Biological Technology Co. Ltd. (Nanjing, China) and aspirin (purity > 98%) was purchased from Solarbio Science & Technology Co., Ltd. (Beijing, China). Evans Blue dye and λ-carrageenan were provided by Sigma (USA). PBS phosphate buffered saline was procured from Leagene Biotech Co., Ltd. (Beijing, China). Formamide was supplied by Macleans Biochemical Technology Co., Ltd. (Shanghai, China).

### λ-carrageenan-induced acute paw edema in mice

The mice were randomly divided into four groups as control (Ctrl), λ-carrageenan (CGN), CGN + aspirin (ASA) (250 mg/kg), and CGN + alloferon-1 (AF) (22 mg/kg), with 5 mice in each group. The control and CGN groups were given 1 ml of 0.9% normal saline subplantar injection in the left hind paw. Positive control groups (CGN + ASA) were pretreated with (250 mg/kg) aspirin by intraperitoneal (IP) injection, and 2 h later, 30 μL of 1% λ-carrageenan was injected into the left hind paw. The needle was inserted from the heel and gently injected under the tendon along the midline of the mice’s sole^10^. In the alloferon-1 groups (CGN + AF), IP injections of alloferon-1 were performed 6 h before λ-carrageenan treatment. The measurements were carried out at 0, 1, 3, 5, and 7 h after λ-carrageenan injection using vernier caliper. The inhibitory effect was determined by using the following formula:

$$\% \,{\text{ Inhibition }} = \frac{{\left( {C_{t} - C_{o} } \right)_{CGN} - \left( {C_{t} - C_{o} } \right)_{treated} }}{{\left( {C_{t} - C_{o} } \right)_{CGN} }} \times 100$$

where ${({C}_{t}-{C}_{O})}_{CGN}$ is the difference in the size of paw at 7 h in CGN mice, and ${({C}_{t}-{C}_{o})}_{treated}$ is the difference in the size of paw at 7 h in mice treated either with the aspirin or alloferon-1.

### Evaluation of paw histopathology by hematoxylin and eosin (HE) staining

Mice were euthanized after injection of λ-carrageenan for 7 h and the left hind paw tissues were fixed with 4% paraformaldehyde (PFA) in phosphate-buffered saline (Sigma-Aldrich) and paraffin-embedded after decalcified with 20% EDTA for 5 days^11^. The paw tissues were cut into five-micrometer thickness sections by microtome. Hematoxylin and eosin (H&E) staining (H-3404, Vector labs, USA) were performed according to the standard protocols. At least five sections from five animals in each experimental group were microphotographed by inverted microscope (Nikon ECLIPSE Ts2, Japan)^12^.

### Assessment of cell counting

Total and differential counts of white blood cell in peripheral blood were determined with a hemocytometer (ADVIA-2120i, Siemens, Germany). After air-drying, blood smears were stained with Hansel Wright dye (Sigma) and washed with phosphate/sodium azide buffer (Sigma) and 95% ethanol. Upon drying, all white blood cell types, including neutrophils, eosinophils, basophils, monocytes, and lymphocytes, can be identified by their appearance under a light microscope.

### Evans blue dye extravasations in λ-carrageenan-induced paw edema

Before tail vein injection, the prepared 0.5% Evans blue PBS sterile solution was put through filter again to remove incompletely dissolved particles. 200 μl of 0.5% Evans blue dye was injected into tail veins of all mice after 7 h of λ-carrageenan injection to measure extravasations of dye in left hind paw tissues. 30 min after injection, mice paws were photographed. Meanwhile, the mice paws were sustained in a 65℃ oven for 48 h to eliminate the difference in moisture. After weighed and crashed, paw tissues were soaked with 500 μl formamide and further heated in a water bath at 55 °C for 24–48 h to extract Evans blue. Afterwards, the formamide/Evans blue mixture was centrifuged at 4000 × g for 10 min. The Evans blue level in the supernatants was determined by its absorption at the wavelength of 630 nm by using a microplate reader.

### Luminex liquid suspension chip detection

After injection of λ-carrageenan for 7 h, blood samples were collected from the retro-orbital plexus, serum separated, and subjected to chip detection. Luminex liquid suspension chip for inflammatory factors detection was performed by Wayen Biotechnologies (Shanghai, China). The Bio-Plex Pro Human Chemokine Panel 23-plex kit (Bio-Rad Laboratories) was applied in accordance with the manufacturer’s instructions. Each sample was assessed in triplicate.

### Western-blot analysis

Protein samples of mice paw tissues were lysed with RIPA buffer and protein was separated in the 12% SDS-polyacrylamide gel electrophoresis (PAGE). The blots were then transferred to a nitrocellulose membrane (Bio-Rad, USA). The membranes were incubated with interleukin-1β (IL-1β) (1:1000, #511369, Zen BioScience, China) or β-actin (1:5000, #250132, Zen BioScience, China) dilutions in TBS containing 1% skim milk. The blots were then incubated with secondary antibodies, HRP-conjugated goat anti-mouse IgGs (Zhongshanjinqiao Co. China) at 1:2000. The experiments were repeated three times with different samples. The blot was cut prior to hybridization with antibodies during blotting. Each blotting gel with 12 loading wells was cut into two equal pieces. Thus, the full-length of the blotting gel included 6 sample-loading wells.

### Statistical analysis

Statistical analysis was carried out using GraphPad Prism 6 (GraphPad Software, San Diego, California, USA). The difference of two groups was confirmed by Student’s t-test and the difference of multiple groups was defined through a one-way analysis of variance (ANOVA) with Tukey’s honestly significant difference test (Tukey’s HSD). All experiments were repeated at least three times independently. The mice samples were randomly collected in all experiments and measurement differences were considered statistically significant at *P* < 0.05.

## Results

### Effect of alloferon-1 on λ-carrageenan-induced paw edema

**Figure 1:**
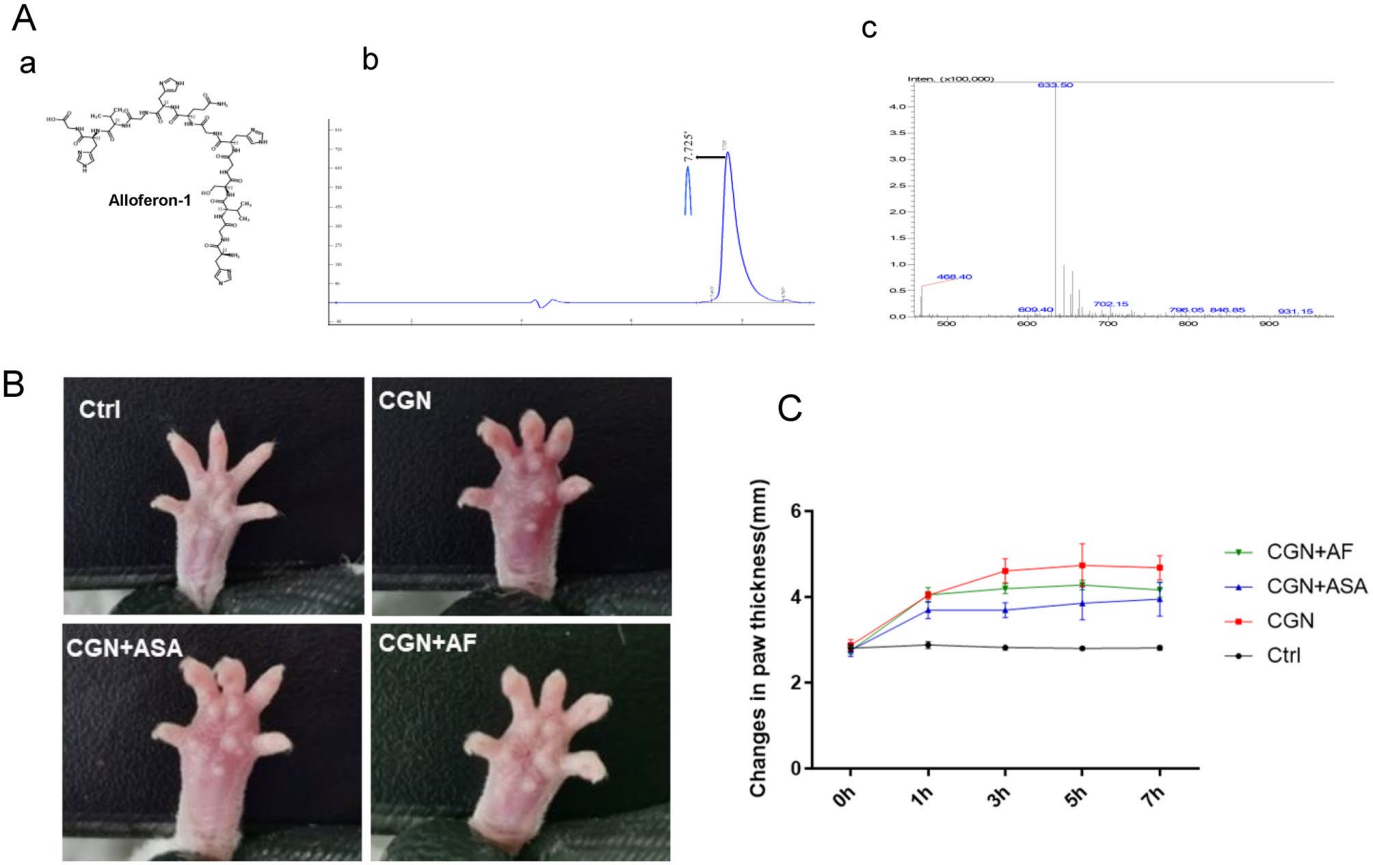
The effect of alloferon-1 on the paw edema in carrageenan-induced mice. Mice were pretreated by intravenous injection with alloferon-1 (22 mg/kg body weight) for 6 h or aspirin (250 mg/kg body weight) for 2 h before subplantar injection of λ-carrageenan. Ctrl: saline solution; CGN: λ-carrageenan; AF: alloferon-1; ASA: aspirin. (**A**) a, Structure of alloferon-1; b, High pressure liquid chromatography (HPLC) detection of synthesized allofereron-1; c. mass spectrometry (MS) analysis of synthesized alloferon-1. (**B**) Appearances of paw edema in each group. (**C**) Measurements of the thickness of paw edema in mice using digital vernier caliper. Data are presented as the mean ± SEM (n = 5). *P < 0.05 were considered as significant difference compared to the control group.

Before experiments, alloferon-1 was synthesized by chemical routes, detected by high pressure liquid chromatography (HPLC) and analyzed by mass spectrometry (MS) (Fig. 1A). In the λ-carrageenan-induced paw edema model, the subplantar injection of λ-carrageenan led to a time-dependent increase in the paw thickness and degree of swelling, as shown in Fig. 1B and C, which reached a maximum at 5 h and sustained elevated thereafter for 24 h. Alloferon-1 was pretreated for 6 h at the concentration of 22 mg/kg of body weight based on the preliminary experiments. Aspirin, as a positive control, was pretreated for 2 h at the concentration of 250 mg/kg of body weight as reported^13^. Both drugs showed negligible effect to paw edema at 0 time. At 1 h after λ-carrageenan-induction, aspirin caused significant reduction of paw edema. Meanwhile, alloferon-1 still showed negligible effect at 1 h after λ-carrageenan treatment. However, after 3 h of λ-carrageenan induction, alloferon-1 significantly reduced the paw edema with a thinner paw and less swelling (*P* < 0.05). As time proceeded, alloferon-1 exhibited a time-dependent inhibitory effect. At 7 h, alloferon-1 reached the maximum reduction effect which was almost the same as the effect of aspirin in inhibition of paw edema. The changes in paw swelling of mice were measured by digital vernier caliper. As shown in Fig. 1C, alloferon-1 and aspirin exhibited 23.03% and 48.82% maximum inhibition rates respectively in comparison to the mice treated with normal saline.

### Histopathological examination and

**Figure 2:**
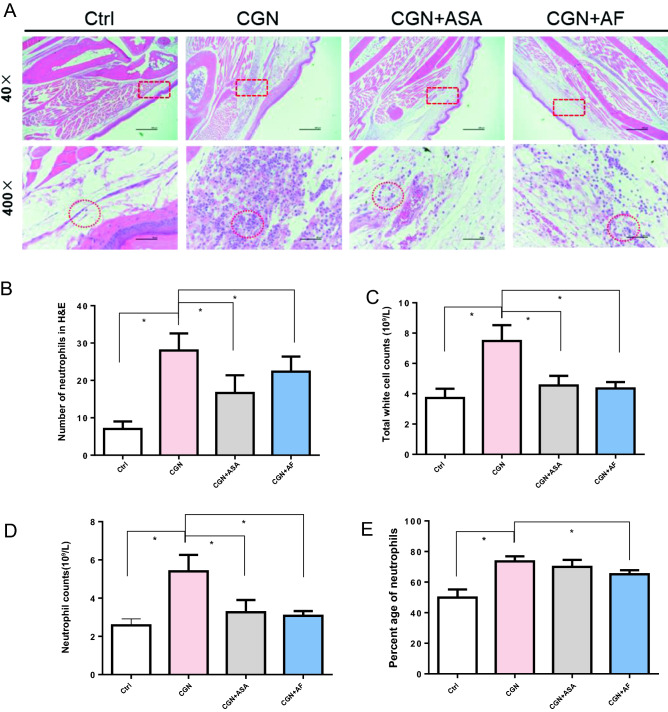
Histopathological examination and assessment of cell counting. (**A**) Histopathological microphotography under microscope with the magnification of 40 × and 400 ×. (**B**) The neutrophils at the position of paw edema were circled with equal-area red dotted lines and was the average number of neutrophils quantified by image J at the magnification of 400 ×. (**C**) Total white cell counts in λ-carrageenan mice blood assessed with hemocytometer. (**D**) The number of neutrophiles. (**E**) The percentage of neutrophils. Numerical data are presented as mean ± SEM (n = 5). *P < 0.05 were considered as significant difference compared to the control group.

The effects of alloferon-1 on the histopathological changes in the paw edema were evaluated by HE staining. As shown in Fig. 2A, in the acute inflammatory response induced by λ-carrageenan, the mice paw exhibited an intense edema, characterized by epithelial and conjunctive tissue blisters with substantial numbers of infiltrated inflammatory cells, most of which were neutrophils. At the position of paw edema, equal areas were selected with red dotted rectangular at 40 × , which were then enlarged at 400 × . The quantification of neutrophil number was performed by counting the cells in equal-area circles with the densest neutrophils. It was showed in Fig. 2B that the inflammation response with infiltration of neutrophils was significantly alleviated by alloferon-1and by aspirin compared to the λ-carrageenan induced mice. Additionally, it was observed that both alloferon-1 and aspirin prevented λ-carrageenan-evoked exudation and influx of neutrophils into mouse pleura and migration of neutrophils into λ-carrageenan-stimulated air pouches in the paw tissue.

Alloferon-1 was found to affect total and differential white cell counts in λ-carrageenan mice blood (Table 1, Fig. 2C–E). In the λ-carrageenan groups, mean total white cell counts was measured as 6.68 ± 2.52 × 10^9^/L, which was significantly increased compared to the control groups (4.55 ± 2.13 × 10^9^/L) (Table 1). In contrast, both alloferon-1 and aspirin showed significant inhibition effect in mean total white cell counts, which was 4.35 ± 0.95 × 10^9^/L and 4.55 ± 1.09 × 10^9^/L respectively (*P* < 0.05) (Fig. 2C). Furthermore, consistent with the histopathological findings, the number of neutrophiles (Fig. 2D, Table 1) and the percentage of neutrophils (Fig. 2E, Table 1) were remarkably decreased in both alloferon-1 and aspirin groups with significance (*P* < 0.05).

**Table 1:** Effect of alloferon-1 on total and differential white cell counts and percentages.

Groups	Ctrl	CGN	CGN + ASA	CGN + AF
White blood cell (× 10^9^/L)	4.55 ± 2.13	6.68 ± 2.52	4.55 ± 1.09	4.35 ± 0.95
Eosinophils (× 10^9^/L)	0.24 ± 0.12	0.23 ± 0.08	0.12 ± 0.02	0.15 ± 0.06
Neutrophils (× 10^9^/L)	2.22 ± 0.99	4.90 ± 1.86	3.27 ± 1.10	2.84 ± 0.68
Neutrophil percentage (%)	49.88 ± 11.97	73.52 ± 7.47	69.97 ± 7.88	65.14 ± 5.85
Lymphocyte percentage (%)	42.18 ± 13.90	21.36 ± 6.42	25.57 ± 7.41	29.40 ± 6.36
Monocyte percentage (%)	1.52 ± 0.31	0.98 ± 0.18	1.07 ± 0.21	1.24 ± 0.25
Eosinophil percentage (%)	5.78 ± 3.04	3.58 ± 1.44	2.77 ± 0.61	3.62 ± 1.37
Basophil percentage (%)	0.36 ± 0.21	0.26 ± 0.09	0.37 ± 0.06	0.26 ± 0.11
Percentage of unstained large cells (%)	0.28 ± 0.18	0.34 ± 0.17	0.27 ± 0.12	0.36 ± 0.25

Data expressed as the mean ± SEM. N = 5 in each group.

### Extravasation of Evans blue dye in paw tissues of mice

**Figure 3:**
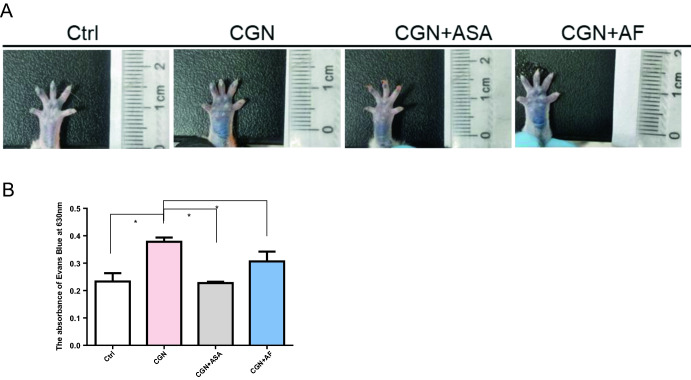
Extravasation of Evans blue in tissues. (**A**) Evans blue injection and observation in paw edema. (B) The Evans blue level in the paw edema tissue was collected and the level was determined by its absorption at the wavelength of 630 nm by using a microplate reader. Numerical data are presented as mean ± SEM (n = 5). *P < 0.05 were considered as significant difference compared to the control group.

Acute inflammation caused by λ-carrageenan can increase the vascular permeability, which can be adjudged by the Evans blue extravasation from the tissues. As shown in Fig. 3, the λ-carrageenan induced paw edema tissues strikingly increased the absorption of Evans blue at the wavelength of 630 nm. Meanwhile, the treatment of both alloferon-1 and aspirin suppressed extravasation of Evans blue dye and exhibited 19.63% and 43.63% inhibition respectively, in comparison to the λ-carrageenan induced paw edema groups.

### The effect of alloferon-1 on the expression of inflammatory factors

**Figure 4:**
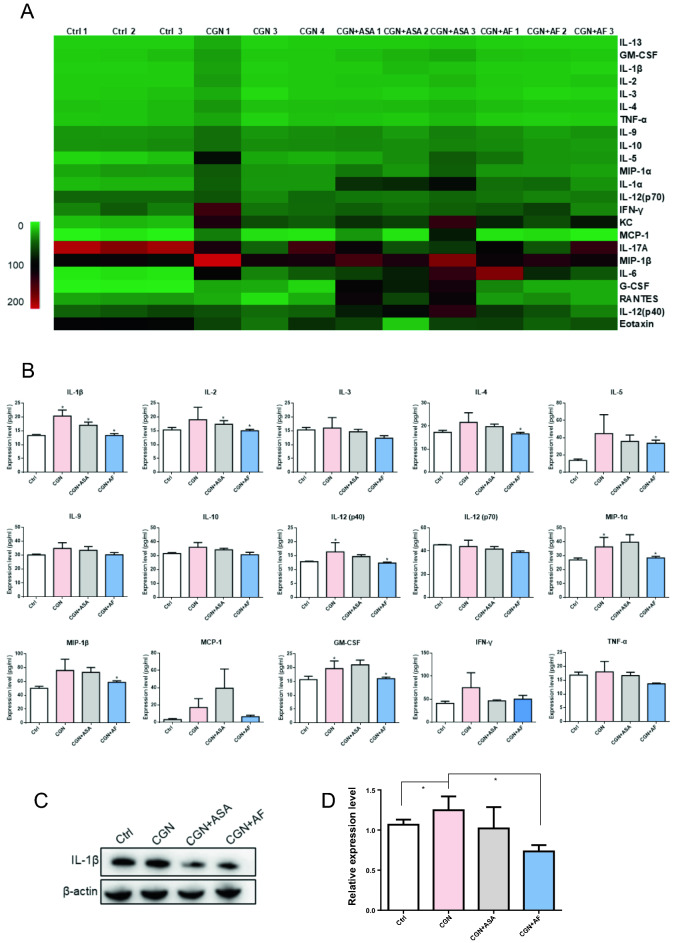
Effect of alloferon-1 on the expression of inflammatory cytokines. (**A**) Heat map analysis of inflammatory cytokines in the serum detected by Luminex liquid cytokine chips (n = 3). The heatmap was generated by Microsoft Excel 2016 (Microsoft Corporation, Redmond, Washington). (**B**) Expression level of cytokines in serum. (**C**) Western blot analysis of IL-1β expression in mice in the serum (n = 3). The original blots are be supplemented in Original images of Fig. 4C. Prior to hybridization with the antibody during blotting, the blotting gel with 12 loading wells was cut into two equal parts. Thus, the full length of the blotting gel was 6 loading wells in total. (**D**) Quantification of the western blot signals with image J. Values are expressed as mean ± SEM. *P < 0.05 were considered as significant difference compared to the control group. Abbreviations: pg/ml, picograms/milliliter; IL, Interleukin; GM-CSF, Granulocyte- macrophage colony stimulating factor; TNF-α, Tumor necrosis factor-α; IFN-interferon-γ; MCP-1, monocyte chemoattractant protein-1; MIP-1, Macrophage inflammatory protein-1; G-CSF, Granulocyte colony stimulating factor.

To further explore the potential mechanism of alloferon-1 in λ-carrageenan-induced inflammation, levels of cytokines in serum were measured by Luminex Liquid Cytokine Chip technology. The expression heat map in Fig. 4A and the quantification histogram in Fig. 4B showed that IL-1β, IL-2, IL-3, IL-4, IL-5, IL-12(P40) was remarkably decreased by alloferon-1 compared to λ-carrageenan-induced paw edema model (*P* < 0.05). Meanwhile, the levels of IL-9 and IL-10 were also slightly decreased by alloferon-1. Additionally, the expression of macrophage inflammatory protein 1α (MIP-1α) and MIP-1β, monocyte chemoattractant protein-1 (MCP-1), granulocyte macrophage-colony stimulating factor (GM-CSF) and TNF-α were downregulated in λ-carrageenan-induced model treated by alloferon-1 compared to λ-carrageenan-induced group. On the contrary, the changes of IL-1α, IL-17A, granulocyte-colony stimulating factor (G-CSF), keratinocyte (KC) and eotaxin did not show apparent changes after the treatment of alloferon-1. IFN-γ showed a slight upregulation by alloferon-1 compared to control but not significant. Moreover, the expression of IL-6 showed slightly upregulation by alloferon-1. Interestingly, the aspirin showed opposite regulatory effects with alloferon-1 in MIP-1α, MCP-1, GM-CSF expression, which were increased after aspirin treatment, suggesting that the anti-inflammatory mechanism of aspirin and alloferon-1 was different.

To validate the expression of cytokine in serum, IL-1β was chosen to be detected by western blot analysis. It was showed in Fig. 4C,D that alloferon-1 decreased the expression of pro-inflammatory factor IL-1β with significance (*P* < 0.05). Collectively, these evidence indicated that alloferon-1 might have an inhibitory effect on the in the λ-carrageenan-induced paw edema by regulation with variety of cytokines.

## Discussion

According to previous studies, alloferons are polypeptides with biological activities including anti-virus, anti-tumor, anti-inflammation, immune regulating, etc.^13,14^ As an immunomodulatory peptide, alloferon-1 has been supported clinically and has been proven to be efficient in patients suffering with herpes simplex virus (HSV) and human papilloma virus (HPV) infections, as well as several Coxsackie viruses^14–17^. However, its anti-inflammation potential needs further preclinical assessment. With the aim of defining the anti-inflammatory properties of alloferon-1, in this study, λ-carrageenan-induced acute paw edema mice model has been chosen. λ-carrageenan-induced paw edema in mice is a well-researched model which has been fully characterized previously^18,19^. According to relevant data, the λ-carrageenan induced inflammation can be divided into three stages, and the first and third stages of inflammation are mainly mediated by E-series prostaglandins, histamine and serotonin^20^. After subcutaneous injection edema, hyperalgesia and erythema appear immediately, which are caused by the action of pro-inflammatory agents-bradykinin, histamine, tachykinin, complement and reactive oxygen species as well as nitrogen substances^21^. Peripheral edema is known to be mainly caused by venous obstruction and increased vascular permeability^22^. Therefore, in this investigation, the changes of paw edema thickness after the injection of λ-carrageenan had been measured to reflect the inflammatory signs. The increase in paw edema by carrageenan lasted only 5–6 h and gradually decreased within 24 h after injection^20^. As an immunomodulatory peptide, alloferon-1 has a relatively slow immunomodulatory effect^7^. Therefore, in this study, alloferon-1 was pretreated to ensure its anti-inflammatory and immunomodulatory effects.

The occurrence of edema causes the increase of vascular permeability, thereby enhancing the efficacy of fluid transport across capillaries and allowing protein-rich liquid to enter the tissue^23^. Inflammatory mediators such as histamine and serotonin cause increase of vascular leakage ^24^. The permeability of Evans Blue dye in the blood vessels of mice is mainly affected by two factors, the internal factors mainly depend on the weight, strain and age of mice, and the external factors are mostly affected by the temperature, humidity and the stress handling of mice^24^. In this study, injections of Evans blue dye into the tail veins of mice were used to evaluate the degree of vascular permeability after the inflammation response caused by λ-carrageenan^25–27^. It was observed that both alloferon-1 and aspirin suppressed extravasation of Evans blue dye in comparison with the λ-carrageenan induced paw edema, suggested that alloferon-1 administration inhibited the vascular permeability in acute inflammation site.

Moreover, the extravasation of the liquid including plasma proteins will cause the leukocytes aggregation and adhesion, which are important features of acute inflammation^22,28,29^. Jane Jeon et al. found that combined application of alloferon-1 and prednisolone to treat asthma significantly reduced the number of eosinophils, macrophages, and neutrophils in bronchoalveolar lavage fluid (BALF) of asthmatic mice induced by ovalbumin (OVA)^7^. In the current study, at 7 h after λ-carrageenan-induction, alloferon-1 significantly inhibited the aggregation and infiltration of neutrophils at the edema location. In addition, the reduction of mean total white cell counts and the number and the percentage of neutrophils by alloferon-1, further confirmed the its anti-inflammatory effect in neutrophils aggregation.

As known, NK cells participate in the development of adaptive immune response through the capability of cytokine production. When activated, NK cells release IFN-γ and TNF-α which in turn reverse the suppression of immune system^30^. IFN-γ and TNF-α are biological factors which kill a large amount of tumor cells and activate lymphocytes (such as T and B lymphocytes)^8,31–36^. NK cells were identified as the peptide pharmacological target responding to alloferon-1 with immediate growth of cytotoxic activity^37^. Alloferon-1 increased the levels of IFN-γ, TNF-α and granular exocytosis produced by NK cells, thus antagonizing cancer cells^5,7–9,37–39^. In addition, previous research has also demonstrated that NF-κB activation is involved in IFN synthesis^16^, and alloferon-1 inhibited the degradation and phosphorylation of inhibitor of κB (IκB) induced by TNF-α in Colo205 colon cancer cells^7^, which provide clues to the basis of alloferon’s capability to stimulate IFN synthesis. In the present study, the expression of IFN-γ and TNF-α were upregulated after λ-carrageenan-induction, which may because that both factors were involved in inflammation reaction. After alloferon-1treament, IFN-γ and TNF-α were detected to be decreased but not significant, which implied an intricate mechanism of alloferon-1 on IFN-γ and TNF-α regulation which needs further investigation. The cytokines IL-2, -12, -15, -18 and -21 are commonly upregulated to increase cellular cytotoxicity of NK cells^40,41^. In this study, IL-1β, IL-2, IL-4, IL-5, IL-12 (P40) was remarkably downregulated by alloferon-1 treatment, which suggested that NK cells mediated acute inflammatory reaction was inhibited by alloferon-1. It has been demonstrated that alloferon-1 prevented inflammatory cell infiltration by down-regulating IL-5 and IL-17 and reduced IgG1 and IgE by inhibiting type 2 T helper auxiliary immune response^7^. IL-5 induces the chemotaxis and activation of integrin CD11b at the molecular level during inflammation and prolongs eosinophil survival by inhibiting apoptosis^42^. In this study, IL-5 was found to be decreased by alloferon-1, which supported the previous findings in λ-carrageenan-induced inflammation.

Alloferon-1 has been reported to play a positive role in the treatment of UVB-induced skin inflammation and ovalbumin-induced asthma^6,43^. The application of alloferon-1 on the UVB-exposed skin of hairless mice showed significantly lowered the increase of skin epithelial thickness of mice, exposed to chronic UVB irradiation^8^. Pro-inflammatory cytokines including IL-1α, IL-1β, IL-6 and IL-18 induced by UVB were reduced by alloferon-1^8^. Recently, it has been reported that alloferon-1 can improve colitis model in mice induced by dextran sulfate sodium (DSS)^9^. Compared with the control groups, the symptoms of edema, epithelial erosion, and immune cell infiltration in the experimental group with the treatment of alloferon-1 were alleviated, and the level of IL-6 in the mouse plasma decreased^7^. In this study, the expression of IL-1β, IL-9 and IL-10 was also downregulated by alloferon-1 in λ-carrageenan-induced inflammatory response. Additionally, we provided experimental evidences that the expression of MIP-1α and MIP-1β, MCP-1 and GM-CSF were reduced by alloferon-1 in λ-carrageenan-treated model. MIP-1α, MIP-1β and MCP-1 are inducible chemokines in response to various proinflammatory stimuli and exhibit a variety of activities including leukocyte chemotaxis^44^. GM-CSF was identified to promote MCP-1-mediated inflammation^45^. We demonstrated for the first time that alloferon-1 downregulated MIP-1α, MCP-1, GM-CSF in immune response, which provides some perspective on anti-inflammatory mechanisms of alloferon-1.

Aspirin exerts its anti-inflammatory effect mainly by interfering with the biosynthesis of cyclic prostaglandins (such as thromboxane A2, prostacyclin, etc.)^46^. Inhibition of prostaglandin leads to alterations in normal protective prostaglandin function, leading to potentially serious side effects such as gastric ulceration, renal failure, impaired platelet function and bleeding complications^47^. Corticosteroids is regarded as the preferred therapeutic drug to treat asthma, however, there are a lot of adverse effects, including inhibition of the hypothalamic-pituitary axis, osteoporosis and opportunistic infections^48^. Originally isolated from bacterial hemolymph^5^, alloferon-1 is consider to be not cytotoxic, immunogenic, mutagenic or carcinogenic, embryotoxic or reproductive toxic, which means it indeed has a particular priority over other chemicals ^17^.

## Supplementary Information

Supplementary Information.
