# Supplementary material for: Alloferon-1 ameliorates acute inflammatory responses in λ-carrageenan-induced paw edema in mice
Source: Sci Rep. 2022 Oct 6;12:16689. doi: 10.1038/s41598-022-20648-z (PMC9537184; doi:10.1038/s41598-022-20648-z)

Original images of Fig. 4C

Replicate 1

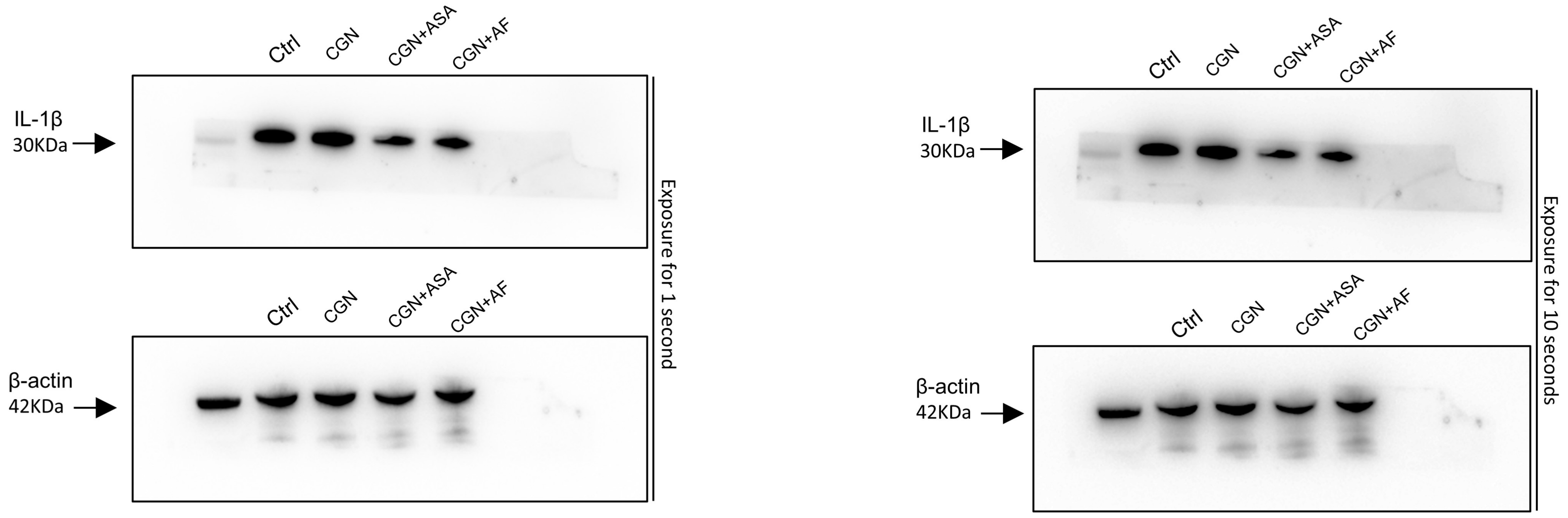

Replicate 2

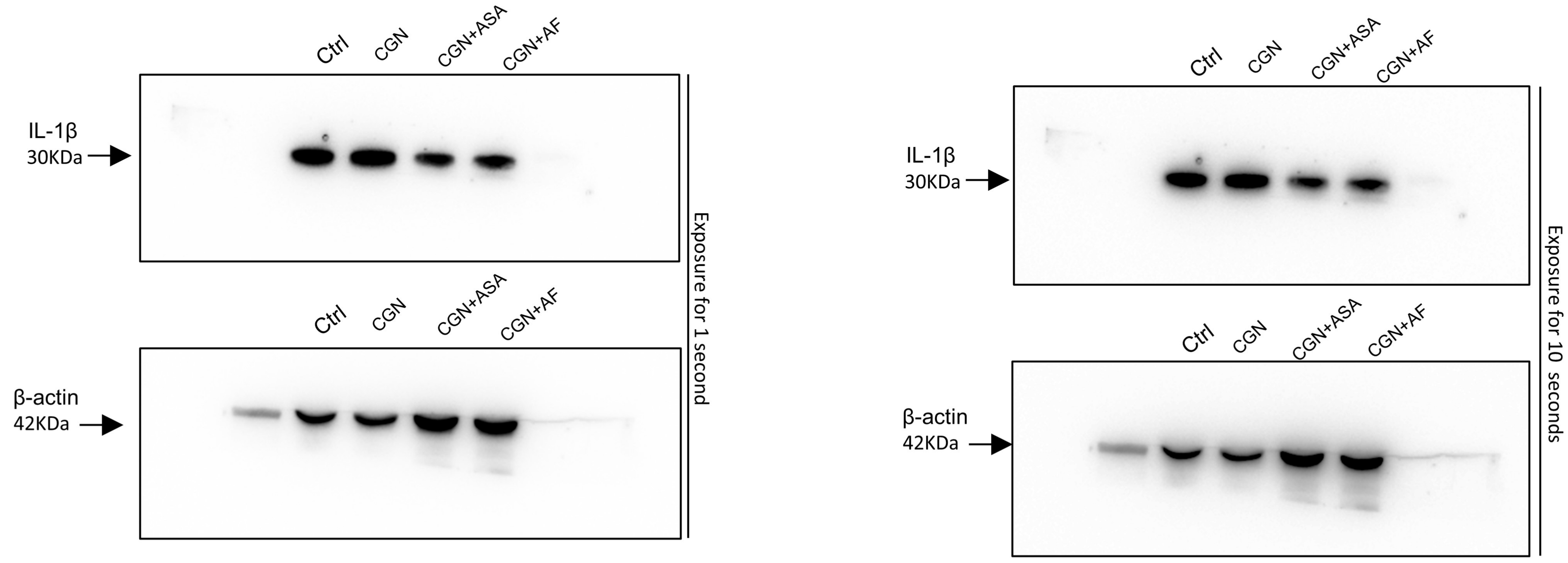

Replicate 3

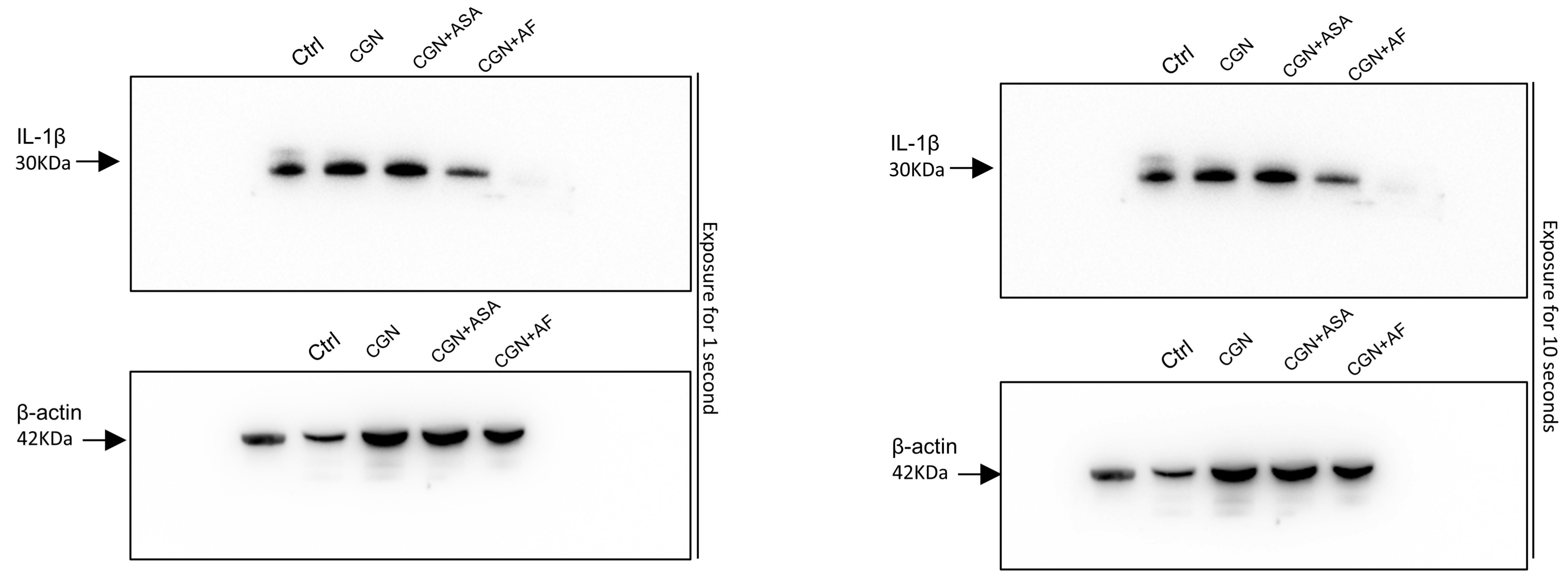

Supplement: Supplementary file 1 — Supplementary Information. [file 41598_2022_20648_MOESM1_ESM.pdf]
